# Mindfulness based stress reduction among substance abuse patients at de-addiction center

**DOI:** 10.6026/973206300181105

**Published:** 2022-11-30

**Authors:** Alfred Solomon D, KalaBarathi S, Vijayaraghavan R, Madhan Krishnan

**Affiliations:** 1 Department of Mental Health Nursing, Saveetha College of Nursing, SIMATS, Thandalam, Chennai, Tamil Nadu, India; 2 Saveetha College of Nursing, SIMATS, Thandalam, Chennai, Tamil Nadu, India; 3 Saveetha Institute of Medical and Technical Sciences, Thandalam, Chennai, Tamil Nadu, India; 4 Faculty of Allied Health Sciences, Chettinad Hospital and Research Institute, Chettinad Academy of Research and Education, Kelambakkam- 603103, Tamilnadu, India

**Keywords:** MBSR, substance abuse patients, salivary cortisol

## Abstract

It is of interest to investigate the effectiveness of a mindfulness-based stress reduction program on stress and salivary cortisol among substance abuse patients. There were 60 drug addicts who were receiving treatment at the addiction centre. Samples are divided into 30 drug abuse patient experimental and 30 substance abuse patient control groups using the simple random sampling approach. Salivary cortisol, a pre-test stress biomarker, was measured and used to analyse the results. The MBSR programme was administered over thecourse of eight weeks, with two 1-hour sessions held each day, with a cap of 15 participants. The biomarker for stress (salivary cortisol) will be obtained once again at the end of the program to assess the post-test level of perceived stress and compare it to the results. Patients showed improvements in stress level (p < 0.05) following the 8-week MBSR program. The mean level of blood cortisol in the experimental group was 18.08 (3.62), which was dropped to 7.54 (1.29) before the intervention. The mean cortisol level in the experimental group differs by 10.54 (3.45) between pre and post intervention following the intervention (p value is < 0.005). Thus, there is a difference between the experimental conditions before and after the intervention. The mean serum cortisol level in the pre-test is 17.30 (2.34) and the mean serum cortisol level in the post test is 17.15 (2.31) in the control group (p value is > 0.005). Data shows that there is a significant difference between the groups. MBSR may be a beneficial intervention for reducing stress, in Patients taking treatment in de addiction centre.

## Background:

 According to the most recent global figures, 36.3 million people, or 13% of all drug users, suffer from drug use disorders. UNODC, World Drug Report 2021.Drugs abuse refers to the excessive use of drugs that tends to activate brain reward system that reinforces behaviours and the production of memories. The Diagnostic and Statistical Manual of Mental Disorders, Fifth Edition includes 10 separate classes of drugs of abuse, which include alcohol, caffeine, cannabis, hallucinogens, inhalants, opioids, sedatives, hypnotics and anxiolytics, stimulants, and tobacco and other substances. These drugs cause such high activation of the reward system that users may neglect previously routine activities. Drugs are chemical molecules that have an effect on both the mind and the body. The precise effects differ across individuals and depend on the drug, dosage, and delivery method. Using any substance, even when used in moderation or under medical supervision, can cause various short-term effects such as changes in appetite, restlessness or insomnia, elevated heart rate, slurred speech, changes in cognitive function, a momentary sense of euphoria, loss of coordination, and so on. Drug abuse can have an impact on parts of a person's life other than their physical condition. Sufferers of substance abuse disorders may exhibit the following symptoms: an inability to quit taking drugs; marital issues; poor career or academic performance; trouble maintaining personal hygiene, Changes in appearance, such as dramatic weight loss; increased impulsivity and risk-taking behaviours; and a loss of interest in formerly enjoyable activities all seem to be signs of a mental health problem. Drug abuse, especially over time, can have a variety of long-term health consequences. Chronic drug use can alter a person's brain structure and function, resulting in long-term psychological impacts such as depression, anxiety, panic disorders, increased aggression, psychosis, hallucinations, and so on. Long-term drug use can also impair memory, learning, and focus. The long-term physical effects of drug usage differ depending on the type of drug and the duration of use. One of the most difficult responsibilities is the treatment of substance abuse. Admission and treatment at a drug rehabilitation facility continue to be useful in the treatment and elimination of substance misuse. Staying in a de addiction treatment centre for an extended period of time may result in psychological changes such as stress, depression, and other psychological illnesses. Low self-esteem, separation from family and friends, economic loss, job loss, future anxiety, and physical health are the stressors largely as a result of substance abstinence and stay in the addiction centre. This study investigated the efficacy of a mindfulness-based intervention in effectively addressing the stress of substance abuse patients at an addiction facility. Mindfulness concerns 'presence of mind' a receptive attentiveness to events and experiences occurring in the present moment, in contrast to a state of mind in which occurrences are habitually filtered through appraisals, evaluations, memories, and beliefs about events and experience [1].Over the last three decades, mindfulness-based interventions have been increasingly included into clinical interventions and wellness programmes to teach individuals how to better regulate stress-related thoughts, emotions, and behaviour. Mindfulness interventions fundamentally tea generalizable approach to stress reduction that is, one can be adapted to a variety of stressful situations over time. Mindfulness interventions have been shown to reduce a variety of psychological symptoms amongst health care providers [2], The purpose of this RCT was to assess the efficacy of a mindfulness-based intervention developed from the empirically supported mindfulness-based stress reduction (MBSR) program [3], for lowering stress and neuroendocrine stress markers, as well as lowering the likelihood of relapse and coping capacities to stop using the substances

A glucocorticoid called cortisol is produced from cholesterol and released into the bloodstream by the adrenal cortex. Most cortisol in blood plasma (65%) binds to corticosteroid-binding globulin with high affinity but poor capacity (transcortin). A total of 30% of cortisol is bound to albumin, while the remaining 3-5% is still in its free, metabolically active state.[4, 5].Therefore, by measuring the amount of cortisol in extracellular fluids, the HPA axis activity during a stress reaction may be identified (blood, urine, saliva). The most often utilised method, which involves measuring blood cortisol levels, has some drawbacks. Additional stress is experienced during the procedure of drawing blood samples from the vein, which might lead to falsely optimistic results [6]. Another shortcoming is that cortisol measured from serum or plasma indicates total cortisol rather than free, physiologically active cortisol. Furthermore, several disorders and medications alter the levels of transcortin and albumin, which alters the level of total cortisol in proportion to free cortisol [7, 8].Due to the aforementioned drawbacks, measuring salivary cortisol levels is receiving more attention today. As a result of diffusion via the basolateral membrane of the salivary gland acini, this hormone accounts for 70% of the non-bound blood cortisol that enters the saliva. Due to its low molecular weight and liposolubility, unbound cortisol diffuses easily through cell membranes [9, 10].Under intense stress, cortisol output rises dramatically [11, 12]. The level ofcortisol secreted in such situations stands in correlation with the intensity of the stress [13].The precise central stress system components that will be engaged in cortisol control during a stressful experience rely on a variety of variables. The kind of stressor is the first of these variables. Physical, biological, and psychological stressors include things like heat, cold, electrical shock, noise, and lack of sleep (e.g. university exams, public appearance, graduation paper defence etc.). The amygdala (AG) is primarily affected by physical stress, while the hippocampus (HC) and perifrontal cortex are affected by psychological stress (PFC). (34Pruessner et al. found that the subjects' HC activation was decreased during psychological stress and came to the conclusion that there was an inverse relationship between HC activation and the cortisol response to stress. On the other hand, different PFC components have a connection to cortisol secretion. Strongly stressed subjects showed increased ventro lateral cortical activity as well as decreased medial, orbitofrontal, and anterior cingulate activity [14, 15 & 16]. Salivary cortisol has the significant benefit that samples can be taken in both the participant's natural environment and in specific conditions outside of the laboratory. Additionally, because the process of collecting saliva is non-invasive, individuals don't experience any added stress [17, 18].Salivary cortisol is a novel technique in the research of acute stress indicators due to its simplicity of collection and potential for wide application.

## Method:

## Participants:

This pilot research examined at a mindfulness-based stress reduction program's feasibility and initial efficacy. There were 60 drug addicts who were receiving treatment at the addiction center. Samples are divided into 30 drug abuse patient experimental and 30 substance abuse patient control groups using the simple random sampling approach. The experimental group members gave their informed consent for the study after being fully informed about it.Salivary cortisol, a pre-test stress biomarker, was measured and used to analyse the results. The study excludessubstance abusers who have just been admitted, substance abusers with a history of seizure disease, serious physical sickness, aggressive conduct, hallucinations, or hostile behaviour.

## Intervention and study materials:

The MBSR intervention is based on a programme created by Jon Kabat-Zinn and colleagues at the Massachusetts Medical Center's Stress Reduction and Relaxation Clinic. Being mindful is having the ability to give your present awareness your whole attention-not just your thoughts.A treatment programme called MBSR teaches participants how to self-regulate their arousal in response to stressful situations or symptoms [19, 20]. By practising meditation, the programme aims to help patients become more conscious of their thoughts and feelings. It also teaches them to pay attention to and monitor their thoughts and feelings in stressful situations to prevent emotional distress. 

 The MBSR programme was administered over the course of eight weeks, with two 1-hour sessions held each day, with a cap of 15 participants. The biomarker for stress (salivary cortisol) will be obtained once again at the end of the program to assess the post-test level of perceived stress and compare it to the results.Patients were asked to keep a diary of their practice time for activities like meditation, walking meditation, and body scans for six days a week for eight weeks after the orientation. Patients were instructed to formally practice for at least 15 to 45 minutes at the first session. The patients went to a 2-hour session on the eighth week, which comprised a practice session and a discussion on nutrition based on the Kabat-Zinn programme.

## Salivary cortisol:

Samples of saliva were collected both before and after the MBSR in both experimental and control group. Samples were obtained by placing synthetic Salivettes under the tongue for two minutes (Salimetrics, State College, PA). The returned samples were immediately frozen at 20 degrees Celsius until the entire sample assay was completed. Samples were then thawed and centrifuged for 15 min at 1500 £ g at 10 degrees C. Cortisol was assayed using the Salimetrics competitive immunoassay method. The inter-assay coefficient of variation (CV) was 6.69%_6.88%, the intra-assay CV was 3.88%_7.12%, and the sensitivity was <0.007 ug/dL.

## Results:

Figure 1 reveals that Before the intervention, the mean level of blood cortisol in the experimental group was 18.08 (3.62), which was dropped to 7.54 (1.29), Following the intervention the mean cortisol level in the experimental group differs by 10.54 (3.45) between pre and post intervention.since P value is < 0.005 It is significant and indicates that there is a difference between the experimental conditions before and after the intervention.In the control group, the mean serum cortisol level in the pretest is 17.30 (2.34) and the mean serum cortisol level in the posttest is 17.15 (2.31).The control group's mean cortisol level varies between pre- and post-intervention by 0.14 (0.84),since the P value is greater than 0.005, the difference between pre and post intervention in the control group is not significant. The p value is less than 0.005 when comparing the posttest values of the experimental and control groups. Demonstrates that there is a significant difference between the groups

## Discussion:

The current pilot study's goal was to advance the field of study and assess how an MBSR programme influenced before psychosocial variables in substance abuse patients receiving treatment at an addiction facility. When someone enters an addiction treatment facility, receives treatment, and achieves relapse, the possibility of relapse looms over them constantly, haunting them like a phantom. As a result, anxiety and stress continue to be a normal part of life even after recovery. Therefore, researchers reasoned that an MBSR programme might be especially beneficial for those who have admitted in de addiction center. The present randomized study was created to investigate if a mindfulness-based psychosocial intervention (MBSR), which is novel for a population of drug abuse patients, would reduce stress in substance misuse patients residing in detoxification facilities [21]. This pilot study discovered that after MBSR, drug misuse patients receiving treatment in addiction treatment facilities reported considerably lower levels of felt stress than the control group. Here, we investigated at cortisol's potential as an objective indicator of change in people who finish the MBSR programme.

## Conclusion:

The hormone cortisol, which the adrenal glands release in reaction to stress, has been demonstrated to be a trustworthy biological indicator of adrenocortical activity and is typically responsive to therapies meant to lessen stress. Cortisol is a viable candidate, when measured with strict protocols, to evaluate the efficacy of programmes meant to lessen stress, like MBSR. Patients receiving treatment at the addiction facility may find that MBSR is a helpful technique for lowering stress.
